# Utilization of a highly adaptable murine air pouch model for minimally invasive testing of the inflammatory potential of biomaterials

**DOI:** 10.3389/fbioe.2024.1367366

**Published:** 2024-04-26

**Authors:** Franziska Woitschach, Marlen Kloss, Sabine Kischkel, Tomáš Macháček, Cindy Reinholdt, Volkmar Senz, Karsten Schlodder, Micha Löbermann, Niels Grabow, Emil C. Reisinger, Martina Sombetzki

**Affiliations:** ^1^ Division of Tropical Medicine and Infectious Diseases, Center of Internal Medicine II, University Medical Center, Rostock, Germany; ^2^ Institute for Biomedical Engineering, University Medical Center Rostock, Rostock-Warnemünde, Germany; ^3^ Department of Parasitology, Faculty of Science, Charles University, Prague, Czechia; ^4^ Biotronik SE & Co. KG, Berlin, Germany

**Keywords:** air-pouch, foreign body reaction, biocompatibility, proinflammatory potential, inflammatory cell infiltration

## Abstract

**Introduction:** The biocompatibility of an implanted material strongly determines the subsequent host immune response. After insertion into the body, each medical device causes tissue reactions. How intense and long-lasting these are is defined by the material properties. The so-called foreign body reaction is a reaction leading to the inflammation and wound healing process after implantation. The constantly expanding field of implant technology and the growing areas of application make optimization and adaptation of the materials used inevitable.

**Methods:** In this study, modified liquid silicone rubber (LSR) and two of the most commonly used thermoplastic polyurethanes (TPU) were compared in terms of induced inflammatory response in the body. We evaluated the production of inflammatory cytokines, infiltration of inflammatory cells and encapsulation of foreign bodies in a subcutaneous air-pouch model in mice. In this model, the material is applied in a minimally invasive procedure via a cannula and in one piece, which allows material testing without destroying or crushing the material and thus studying an intact implant surface. The study design includes short-term (6 h) and long-term (10 days) analysis of the host response to the implanted materials. Air-pouch-infiltrating cells were determined by flow cytometry after 6 h and 10 days. Inflammation, fibrosis and angiogenesis markers were analyzed in the capsular tissue by qPCR after 10 days.

**Results:** The foreign body reaction was investigated by macroscopic evaluation and scanning electron microscopy (SEM). Increased leukocyte infiltration was observed in the air-pouch after 6 h, but it markedly diminished after 10 days. After 10 days, capsule formations were observed around the materials without visible inflammatory cells.

**Discussion:** For biocompatibility testing materials are often implanted in muscle tissue. These test methods are not sufficiently conclusive, especially for materials that are intended to come into contact with blood. Our study primarily shows that the presented model is a highly adaptable and minimally invasive test system to test the inflammatory potential of and foreign body reaction to candidate materials and offers more precise analysis options by means of flow cytometry.

## 1 Introduction

Globally, there is a constantly increasing demand for medical devices (Tzoulaki et al., 2016; Geelhoed et al., 2017). The market for medical devices is estimated to grow at a compound annual growth rate (CAGR) of 5.5% until 2029 (Fortunebusinessinsights, 2022). In all areas of application, a high degree of material biocompatibility is required to ensure functionality and avoid rejection reactions (Helmus et al., 2008). The function and longevity of a medical device is determined not least by the patient’s immune response, technical functionality and resistance to infections (Londono and Badylak, 2015; Kutner et al., 2021). Any implantable medical device triggers a non-preventable foreign body reaction (FBR) upon implantation by provoking innate immune responses (Anderson et al., 2008; Corradetti, 2017; Wei et al., 2019). The FBR is characterized by a number of events. First, the implant becomes covered with plasma proteins, known as the Vroman effect (Hirsh et al., 2013) which occurs in the first few seconds after implantation and is of particular importance for implants in contact with blood. This adsorbed protein layer can in turn lead to adhesion/activation of platelets, inflammation and finally, in the worst case, implant-induced thrombosis (Brash et al., 2019; Frutiger et al., 2021). Over the next few hours, inflammatory cells are recruited, including neutrophils, macrophages and fibroblasts, which release cytokines such as TNF-α, IL-10 and growth factors (Anderson et al., 2008). The presence and polarisation of macrophages play an important role in the regulation of FBR (Wei et al., 2019; Mukherjee et al., 2020). Similar to pathogen defense, macrophages attempt to degrade and eliminate any foreign agent (Corradetti, 2017; Carnicer-Lombarte et al., 2021). When degradation and phagocytosis fail, fibroblasts encapsulate the material and form a physical barrier to isolate it from the rest of the body, which remains in place as long as the device remains in the patient. In this context, long-term implantations pose a particular challenge, as encapsulation prevents interaction between the implant and the target tissue. To avoid the consequences of a foreign body reaction, materials with high biocompatibility are needed.

Medical silicones are such materials with a very high biocompatibility and are therefore suitable for medical applications. However, even with medical silicones, the formation of a fibrous capsule cannot be sufficiently controlled and suppressed, demonstrated by the evaluation of long-term biocompatibility in the permanent implantation of medical silicones for breast implants (since 1963) and intraocular lenses (since 1978) (Auffarth and Apple, 2001; Maxwell and Gabriel, 2009; Zeplin et al., 2014). Current implant research is therefore focused on modifying implant surfaces to prevent non-specific protein adsorption and control the inflammatory response. One way to improve biocompatibility is to create a non-fouling surface that prevents non-specific binding of proteins and bacteria (Shi et al., 2008; Zhang et al., 2008). Surface modifications can be achieved by chemical, mechanical, biological and physical methods (Yu et al., 2014; Fabbri and Messori, 2017). In the physical context, surface modification with zwitterions is a promising approach to reduce the initial attachment of proteins and subsequently the attachment of bacteria, for example, through the formation of a hydration shell or steric hindrance (Azari and Zou, 2013). One of the best characterized zwitterions is 2-methacryloyloxylethyl phosphorylcholine (MPC). Tan et al. used MPC grafts to improve the biocompatibility of the intraocular lens. They implanted the modified lens in rabbits and found that inflammation and encapsulation decreased significantly (Tan et al., 2017).

In the present study, two thermoplastic polyurethanes (TPU) and two modified liquid silicone rubbers (LSR) were compared. The polymer modifications with polymethyl methacrylate-2-methacryloyloxyethyl phosphorylcholine (PMMA-MPC) and polysulfonate (PSU) were tested in a mouse air-pouch model. TPUs are widely used in medical field, it represents the state-of-the-art material, with its excellent physical and mechanical properties, including durability, tear strength, and stress and puncture resistance. Additional elastomeric and flexibility properties make this material a more versatile choice, as well as the key features excellent biocompatibility, transparency and outstanding environmental resistance (including various gases and chemicals). PSU is an anti-adhesive polymer used for membranes for ultrafiltration due to its hydrophobic properties, high pH and temperature resistance (Maheswari et al., 2013; Yunos et al., 2014). In our previous work, these materials have already been investigated with regard to their effect on the interaction with innate immune cells (Woitschach et al., 2021a; Woitschach et al., 2021b; Woitschach et al., 2022).

Herein, we have modified the murine air-pouch model for simple and minimally invasive testing of materials in the form of micro-stents. The air-pouch model is an *in vivo* model in which repeated subcutaneous injections of air lead to the formation of a defined, well-perfused connective tissue sheath. By inserting the micro stents, a specific immune response will occur, which can be mapped using the wound fluid, the exudate. This animal model can be utilized to study local inflammation without systemic effects (Duarte et al., 2012). The implant specimens are easy to insert and exudates can be collected and comprehensively analyzed. It is a simple, low-burden and reproducible mouse model, in which biomaterials can be observed over long periods of time, allowing the analysis of the inflammatory and tissue activating properties of the materials (Fehrenbacher and McCarson, 2021; Peng et al., 2021).

In the present study, the air-pouch model was used for short- and long-term analyses to evaluate implant materials in terms of inflammatory potential and foreign body response by analyzing the recruited cells in the exudate using flow cytometry, the inflammatory response using microscopic and cellular analysis methods, and the encapsulation of the implants using microscopy.

## 2 Materials and methods

### 2.1 Ethical statement

All animal experiments were performed in strict accordance with the regulations of the German Society for Laboratory Animal Science and with the European health guidelines issued by the Federation of Laboratory Animal Science Associations. The protocol was approved by the local committee on animal care and use (7221.3-1-068/20). Full efforts were made to minimize animal suffering.

All tests, including animal experiments, were carried out in accordance to Good Laboratory Practice (GLP).

### 2.2 Mouse air-pouch model and implantation procedure

**FIGURE 1:**
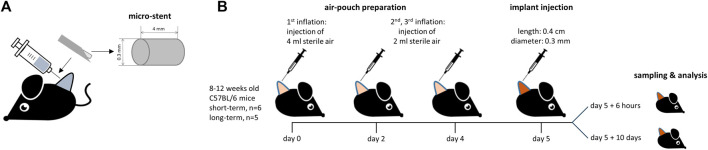
**(A)** Implant and **(B)** air-pouch study design.

Female C57BL/6 mice aged 8–12 weeks were purchased from Janvier Labs (Saint Berthevin, France). All mice were maintained on a standard diet with food and water *ad libitum* and a 12-h light-day-night rhythm. Under isoflurane anesthesia (0.6 mL/L oxygen and 2.5% isoflurane), 4 mL sterile air was injected into the shaved skin at the level of the scapulae of each mouse to create a dorsal subcutaneous air-pouch. The air-pouches were inflated with 2 mL of sterile air on days 2 and 4. On the fifth day, the biomaterial was implanted in a minimally invasive procedure under isoflurane anesthesia using an in-house, easy-to-use application system (Figures 1A, B). This consisted of a 5 mL syringe (Discardit; Becton Dickinson, United States) and a 21 ½-G cannula (MicrolanceTM 3; Becton Dickinson, United States). The implant was inserted into the cannula under sterile conditions before being inserted into the air-pouch. The implants were left in the mice for 6 h to analyze the short-term response or for 10 days to analyze the long-term response (short-term: n = 6 animals per experimental group and time, long-term: n = 5 animals per control group and time). Animals receiving phosphate buffered saline (PBS) served as controls.

### 2.3 Implant materials and controls

Test specimens in the form of micro-stents made of two thermoplastic polyurethanes (TPU) and liquid silicone rubber (LSR) with and without surface modification were used (Biotronik, Berlin, Germany): thermoplastic polyurethane 55 = P55, thermoplastic polyurethane 80 = P80, LSR + polymethyl methacrylate-2-methacryloyloxyethyl phosphorylcholine = PMMA-MPC (Tan et al., 2017) and LSR + polysulfone = PSU. The test specimens with surface areas of approx. 0.3 mm and a length of 4 mm (Figure 1A) were subjected to a sterilization process with ethylene oxide (ETO).

### 2.4 Blood analysis

After 6 h (short-term experiment) or 10 days (long-term experiment) the animals are deeply anaesthetized by i. p. injection of ketamine (90–120 mg/kg) and xylazine (6–8 mg/kg) and released by a final retrobulbar blood sampling and cervical dislocation. Subsequently, the air-pouch exudate and capsular tissue were collected. The collected blood was analyzed for the percentage composition of inflammatory cells in a hematological analyzer HM5 vet; SCIL animal care company GmbH (Viernheim, Germany).

### 2.5 Flow cytometry

To analyze recruited inflammatory cells the air-pouches were rinsed with 3 mL of ice-cold PBS, softly kneaded, and exudates were collected. The liquids obtained were centrifuged (5 min, 350 ×*g*, 4°C). Staining with the Zombie Red Fixable Viability Kit (1:2000, BioLegend) was performed to exclude dead cells. After washing (3% FBS/PBS), cells were incubated (20 min, 4°C) with the following fluorophore-labeled antibodies (BioLegend, if not stated otherwise) to identify major leukocyte populations: anti-CD16/CD32 (1:100, clone 93), anti-CD45-APC-Cy7 (1:150, 30-F11), anti-CD11b-APC (1:800, M1/70), anti-CD11c-AF488 (1:400, N418), anti-Ly6G-APC-Cy7 (1:150, 1A8), anti-SiglecF-PerCP-Cy5.5 (1:100, E50-2440, BD Biosciences), anti-F4/80-PE-Cy7 (1:100, BM8), anti-I-A/I-E (MHC II)-BV421 (1:120, M5/114.15.2), anti-CD19-AF488 (1:150, 6D5), anti-CD3ε-APC (1:120, 145-2C11), anti-CD4-PerCP-Cy5.5 (1:200, RM4-4), anti-CD8a-PE-Cy7 (1:600, 53-6.7), anti-CD152 (CTLA-4)-PE (1:100, UC10-4B9). Fluorescence minus one control was used for CTLA-4, CD11c, and MHC II. After staining, the cells were fixed with 2% formaldehyde (10 min, 4°C), washed, and measured by FACSAria IIIu (BD). The data was analyzed in FlowJo v.10.7 (BD). For further information on the antibodies and their targets, see Table 1.

**TABLE 1:** Antibodies used for flow cytometry in this study and their target function.

Antibody/marker	Target/function
Zombie Red	Live/dead cell staining
anti-CD16/CD32	Differentiation of neutrophils, monocytes, macrophages and t-cells
anti-CD45-APC-Cy7	Leukocyte differentiation
anti-CD11b-APC	Microglial cell markers
anti-CD11c-AF488	Dendritic cells
anti Ly6G-APC-Cy7	*In vivo* function of neutrophils
anti-SiglecF-PerCP-Cy5.5	sialic acid-binding Ig superfamily receptor that is highly expressed on eosinophils
anti-F4/80-PE-Cy7	mouse macrophages
anti-I-A/I-E (MHC II)-BV421	MHC Class II, antigen presentation
anti CD19-AF488	B-cell development
anti-CD3ε-APC	T-cell differentiation
anti-CD4-PerCP-Cy5.5	T- cell differentiation (T-helper cells)
anti-CD8a-PE-Cy7	T-cell differentiation (cytotoxic T-cells)
anti-CD152 (CTLA-4)-PE	T-cell differentiation (regulation of immune responses)

### 2.6 Morphological analysis of implant encapsulation

To investigate encapsulations, the implants were recovered from the air-pouches using a stereomicroscope. For scanning electron microscopy (SEM) samples were fixed in 2.5% glutaraldehyde, dehydrated in a descending ethanol series, and subjected to critical point drying. The samples were dried with hexamethyldisilane and then gold-plated with a gold sputtering unit.

After 10 days, the implants and surrounding capsules were examined macroscopically with a binocular. Images were taken and the size of the tissue capsules was evaluated using ImageJ software (v1.47v; National Institute of Health).

### 2.7 Gene expression analysis

Total RNA was isolated from snap-frozen capsule tissue (RNeasy Plus Mini Kit, Qiagen, Germany) and reversely transcribed into cDNA using High-Capacity cDNA Reverse Transcriptase Kit (ThermoFisher, Germany) according to the manufacturer’s instructions. QPCR was performed using the TaqMan Gene Expression Assays, see Table 2 (ThermoFisher, Germany). Cycling was performed on the Quant Studio 3 under the following reaction conditions: 50°C for 2 min followed by 95°C for 10 min, 45 cycles at 95°C for 15 s, and at 60°C for 1 min. Gene expression values were normalized to the endogenous reference gene gapdh (Rodent gapdh control reagent, ThermoFisher, Germany) and presented as normalized expression values relative to PBS controls.

**TABLE 2:** List of TaqMan assays (ThermoFisher) used in this study.

Gene Symbol	Gene name	Assay ID
*tgf-β1*	transforming growth factor, beta 1	Mm01178820
*col1a2*	collagen, type I, alpha 2	Mm00483888
*il-6*	interleukin 6	Mm00446190
*tnf-α*	tumor necrosis factor α	Mm00443258
*ace*	angiotensin I converting enzyme (peptidyl-dipeptidase A) 1	Mm00802048
*thbs1*	thrombospondin 1	Mm01335417

### 2.8 Statistics

Statistical analysis was performed using GraphPad Prism 9 (GraphPad Software, La Jolla, CA, United States). Values are expressed as mean +SEM. Normal distribution was tested using the D'Agostino and Pearson omnibus normality tests. Normally distributed samples were compared using ANOVA followed by Bonferroni *post hoc* test. Non-normally distributed samples were compared using the Kruskal-Wallis test followed by a Dunn’s *post hoc* test. In all statistical analyses, *p*-values <0.05 were considered significant. *p < 0.05, **p < 0.01, ***p < 0.001, n. s., not significant.

## 3 Results

### 3.1 The insertion of the test specimens into the air-pouch leads to a modest change in the blood cell composition

**FIGURE 2:**
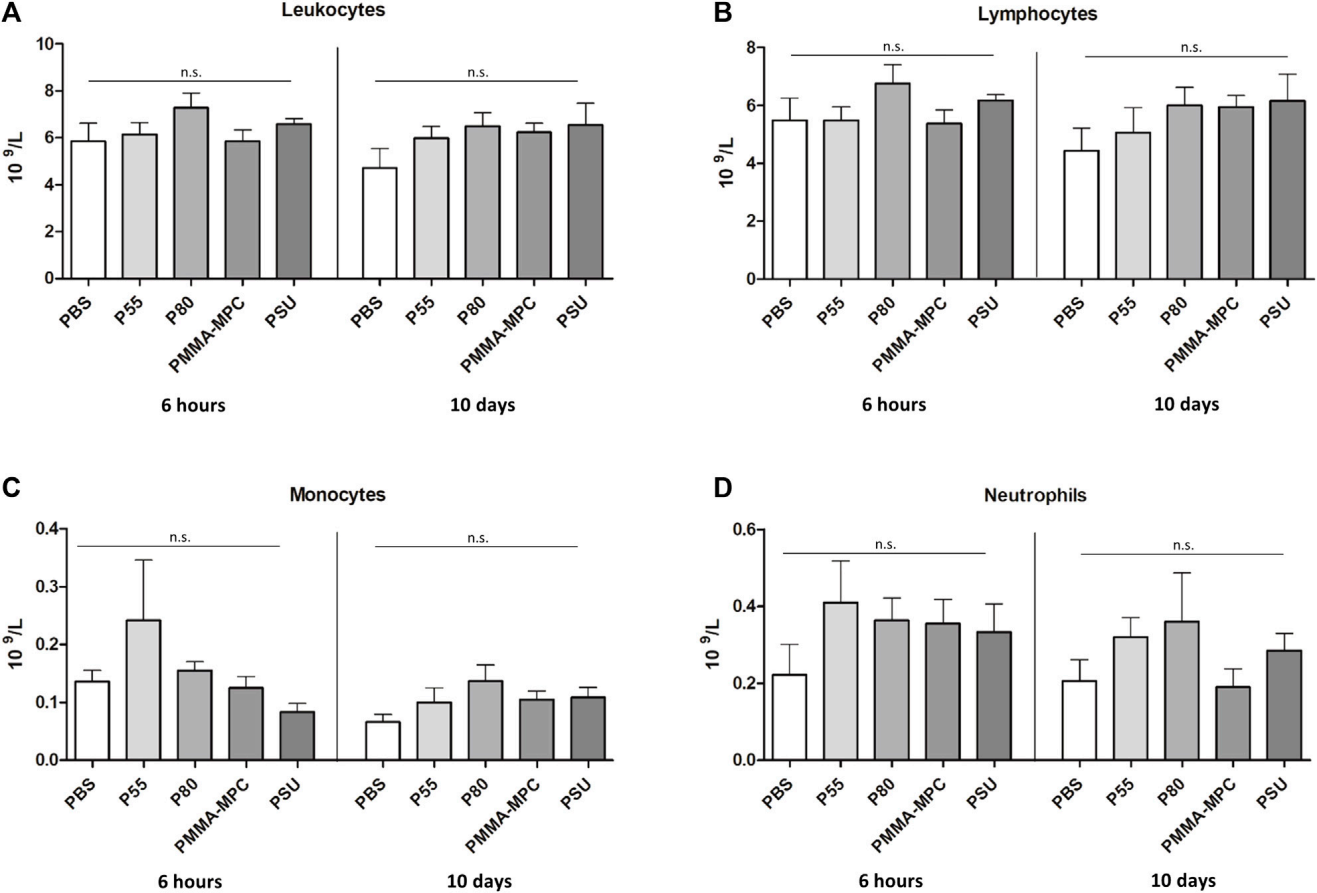
The insertion of the test specimens into the air-pouch leads to a modest change in the blood cell composition. The composition of the blood cells changes marginally depending on the material samples tested in the air-pouch. **(A)** Number of leukocytes, **(B)** lymphocytes, **(C)** monocytes and **(D)** neutrophils are shown after 6 h and 10 days respectively. Data are represented as mean ± SEM of 6 biological replicates per group. For all statistical analyses, *p* values <0.05 were considered significant, differences in this figure were not statistically significant.

In order to investigate whether an inflammatory influence of the test specimens in the air-pouch could also be seen systemically, the blood cells were quantified. Although no statistical significance was achieved, it was shown that polyurethane 80 led to a short-term increase in leukocytes and lymphocytes. Similar to polyurethane 55, polyurethane 80 also leads to a slight increase in monocytes in the blood. An increase in neutrophils is caused by all test samples. After 10 days, there is an increase in leukocytes, lymphocytes, monocytes and neutrophils in all tested materials (with the exeption of PMMA-MPC) compared to the injection of PBS (Figure 2).

### 3.2 The recruitment of inflammatory cells into the air-pouch can be mapped in a material- and time-dependent manner

**FIGURE 3:**
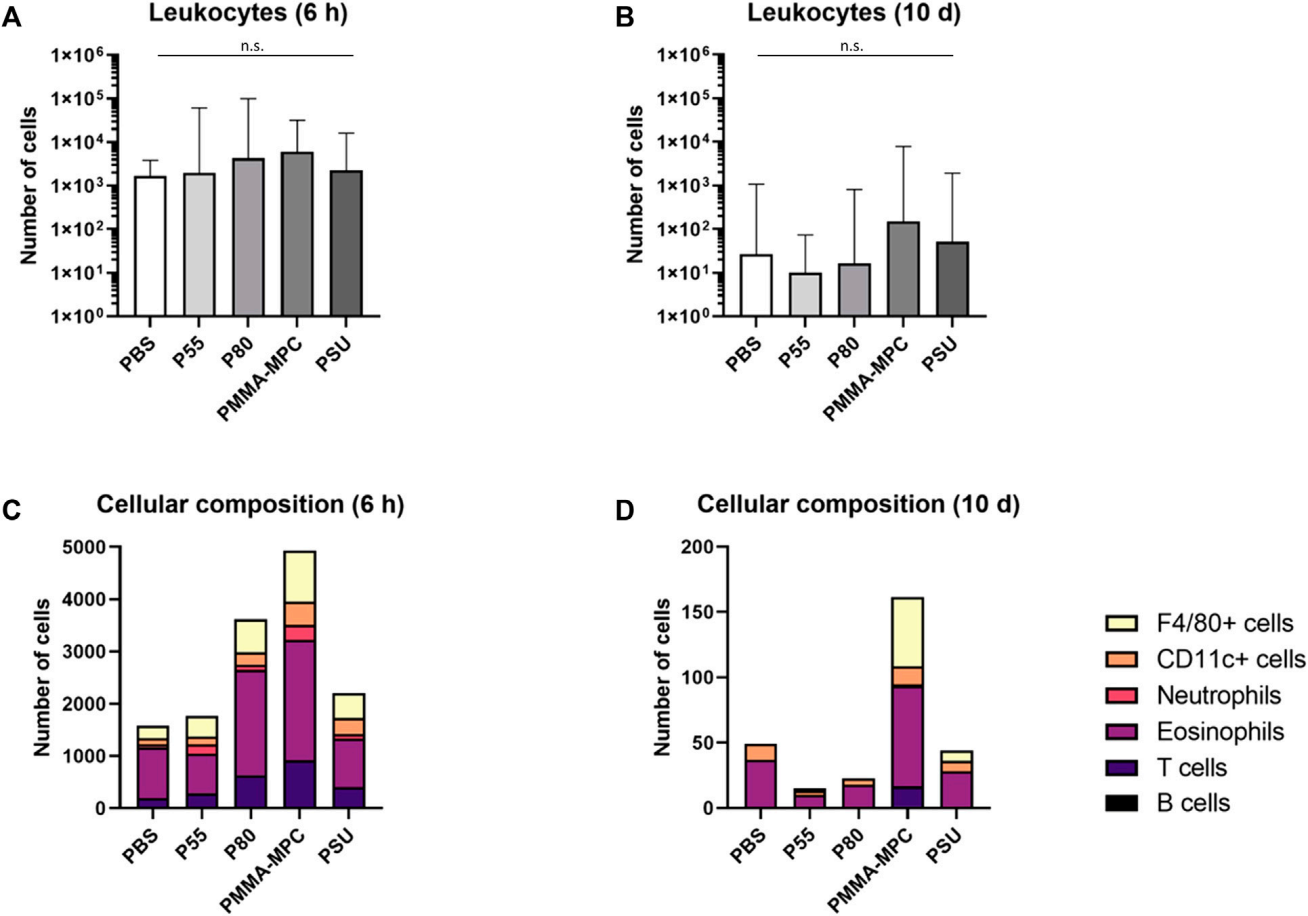
The recruitment of inflammatory cells into the air-pouch can be mapped in a material- and time-dependent manner. **(A,B)** Ten days after application of the materials, the recruitment of leukocytes decreases compared to the short-term test (6 h). Shown are medians with 95% confidential intervals, n = 5-6 per group. **(C,D)** The composition of the leukocyte population in the air-pouches 6 h and 10 days after application of the materials is shown. The medians are presented without error bars to keep clarity, n = 5-6 per group. For all statistical analyses, *p* values <0.05 were considered significant, differences in this figure were not statistically significant.

To investigate the local inflammatory reaction in response to the implantation of the test bodies, the cell composition of the air-pouch exudates was analyzed by flow cytometry. It was found that the air-pouch exudates of the animals from the short-term experiment contained more leukocytes than those from the long-term experiment (Figures 3A, B). Regarding the composition of the inflammatory cell infiltrate, PMMA-MPC was conspicuous as it tended to attract more cells than the comparison materials. An above-average number of eosinophilic granulocytes and F4/80+ cells (mostly macrophages) were detected in all exudates compared to the PBS control after 6 h (Figure 3C). In contrast, about 30 times fewer leukocytes were isolated from the air-pouch exudates of all groups at a later time point (10 days) (Figure 3B). No differences were detected with regard to antigen-presenting MHC-II^+^ and/or CD11c^+^ cells and immunoinhibitory CTLA-4^+^ T cells.

### 3.3 The air-pouch model enables the analysis of inflammatory, fibrotic and angiogenic processes during the encapsulation of the implant

**FIGURE 4:**
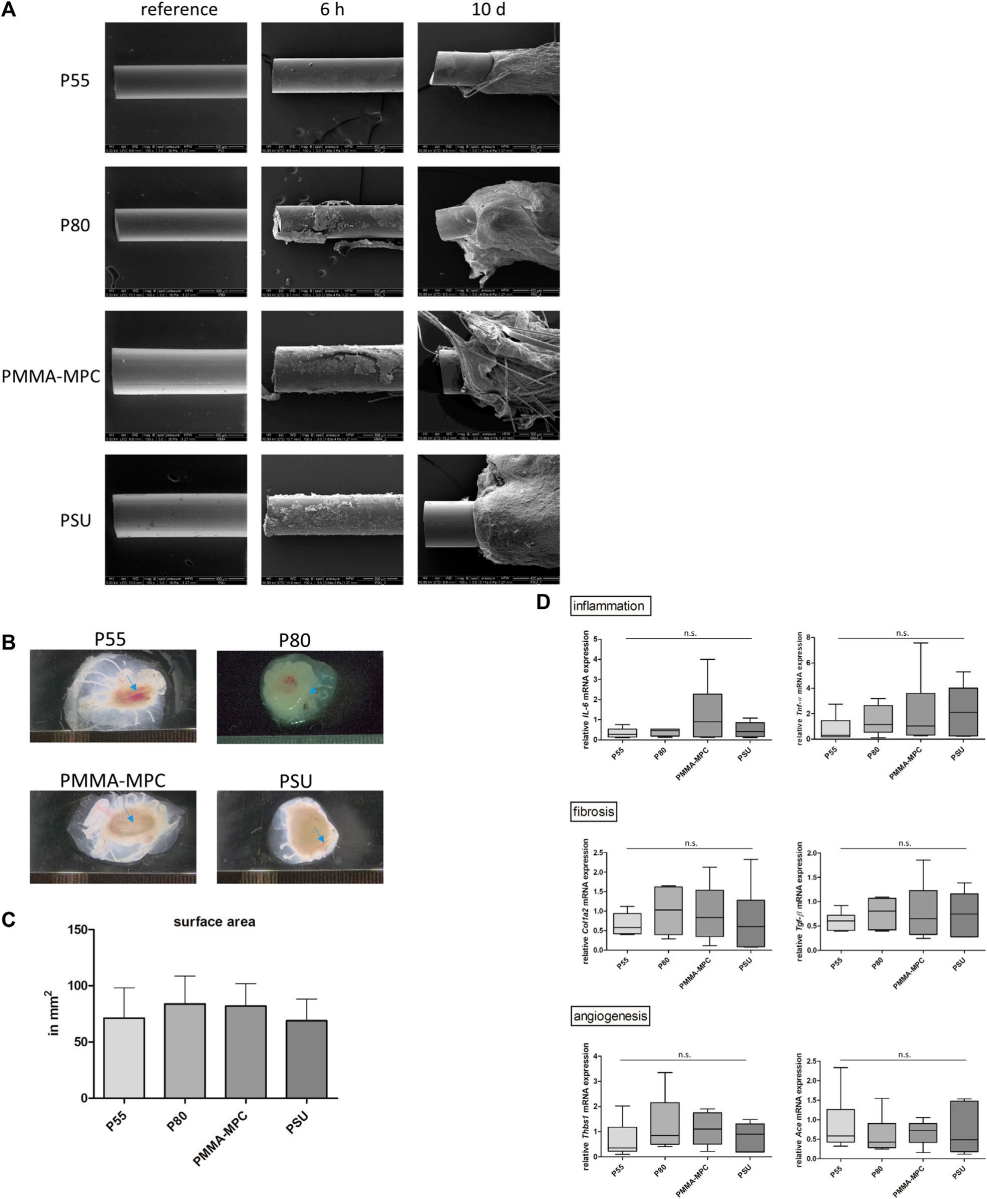
The air-pouch model enables the analysis of inflammatory, fibrotic and angiogenic processes during the encapsulation of the material. **(A)** Foreign body reaction originating from the different implant material analyzed by scanning electron microscopy after 6 h and 10 days. Magnification, 100 × **(B)** Macroscopic presentation of the different encapsulated material implants (blue arrows). **(C)** Quantification of the tissue capsule surface area using ImageJ software showed no differences in size. **(D)** Analysis of relative expression of proinflammatory genes, profibrotic genes and angiogenic genes in the tissue capsule around the implants after 10 days. Data are normalized to the endogenous reference gene *gapdh* (rodent *gapdh* control reagent) and are presented as normalized expression values relative to PBS controls, shown as mean ± SEM of n = 6 per group. Data are represented as mean ± SEM of 5-6 biological replicates per group. For all statistical analyses, *p* values <0.05 were considered significant, differences in this figure were not statistically significant.

In order to evaluate both, the early and late host cellular response at the implant surface, scanning electron microscopy (SEM) of the implants was performed after 6 h and 10 days. The results of the SEM were obtained at both time points. Increasing encapsulation of the implants was observed at both time points. After 6 h, the first cellular deposits were visible on the surface of all materials, while after 10 days, multilayer encapsulation had formed, with P55 being the least affected (Figure 4A).

Ten days after implantation of the materials, the extent of encapsulation of all implant materials in the air-pouches was measured (Figures 4B, C). There were no differences in the capsule sizes of the different materials. However, for P55 and PSU, the diameter of the capsules was slightly smaller on average than for P80 and PMMA-MPC (Figure 4C).

Defined parts of the capsular tissue were used to analyze the proinflammatory genes *il-6* and *tnf-α*, the profibrotic genes *col1a1* and *tgf-β*, and the angiogenic genes *thbs 1* and *ace*. Downregulation of *il-6* expression was observed in P55, P80 and PSU compared to the PBS control (Figure 4D). PSU showed a slight increase in *tnf-α* expression compared to PBS (Figure 4D). The expression of the profibrotic genes *col1a1* and *tgf-β* was slightly increased in the capsules of all materials (Figure 4D). The lack of or low expression of *thbs* and *ace* in the tissue capsules of all specimens also indicates an attenuated foreign body response by the implanted materials (Figure 4D).

## 4 Discussion

The primary objective of this study was to compare the materials used in this particular model in terms of their biocompatibility *in vivo*. And we were able to show that the air sac exudate in particular provides valuable analysis material, for example, for FACS analyses, which conventional tissue analyses do not provide. We were able to demonstrate that the murine air-pouch model is suitable for investigating the biocompatibility of the various materials tested, with particular attention to their inflammatory potential and capsule formation. The investigated polyurethanes and modified silicones in the air-pouch model cause foreign body reaction (FBR) with capsule formation, but show only minor signs of tissue inflammation. The use and range of applications of biomaterials in medicine continues to increase (Fortunebusinessinsights, 2022). Along with this, research into the interaction between host cells and implants is becoming increasingly important. As one of the main factors for the loss of functionality of an implant as a whole, FBR is a critical issue in implant surgery (Lambris et al., 2015; Zhang et al., 2021). In this study, we used common thermoplastic polyurethanes and novel modified medical grade silicones to investigate FBR including inflammatory cell recruitment and cell adhesion to the implant materials in an air-pouch model. In the air-pouch model, a sterile subcutaneous cavity is formed into which the biomaterial can be inserted. For the duration of the trial, the cavity fills with a wound-healing-like fluid that allows cells to migrate. Few studies have attempted to investigate the mechanisms of the inflammatory response to different materials, organisms and substances using the air-pouch model (Chiappini et al., 2012; Lovric et al., 2018; Sombetzki et al., 2018). For example, Lovric et al. (Lovric et al., 2018) compared intact suture material with suture particles in the air-pouch model by inserting the material through an incision. The suture particles caused a stronger inflammatory response with more multinucleated giant cells and higher metalloproteinase (MMP) expression.

The present study differs from other air-pouch model studies in two aspects: 1) a complete piece of material of defined size is used, 2) the material is applied minimally invasively with a syringe, avoiding a skin incision and thus problems that may be associated with wound healing. With the model presented, it is possible to easily perform the FACS analyses of the inflammatory exudate. Similarly, analysis of soluble inflammatory markers can be performed using the air-pouch fluid. Thus, the model has many advantages, e.g., compared to the examination of subcutaneously introduced material. In addition, implants in blood contact can also be examined with this model, as cell recruitment into the air-pouch is comparable to cell recruitment from the bloodstream.

In previous *in vitro* studies, we have shown that the zwitterionic material PMMA-MPC reduces biofilm formation of *Staphylococcus aureus* ATCC 35556 (Woitschach et al., 2022). Furthermore, PMMA-MPC induces a higher inflammatory response in monocytes (Woitschach et al., 2021b). In addition to resistance to infection, the tendency to inflammation is also a critical quality feature of implants. Therefore, we used the air-pouch model to investigate the inflammatory potential of PMMA-MPC. In the current *in vivo* study, PMMA-MPC seems to recruit more leukocytes into the exudate of the air-pouch compared to all other investigated materials (Figures 2A, C), confirming the increased inflammatory response *in vitro* (Woitschach et al., 2021b). For the thermoplastic polyurethane P55, only low cell recruitment (Figure 2), low cell attachment (Figure 3) and the lowest expression levels for genes involved in processes such as inflammation, fibrosis and angiogenesis were observed (Figure 4D). Our results suggest that PMMA-MPC is the material that effectively prevents biofilm formation, but also has the highest inflammatory potential *in vitro* and elicits the strongest cellular response *in vivo*. P55, on the other hand, did not show any effects against biofilm formation, but elicited only a modest cellular response *in vitro* and *in vivo*.

2-Methacryloyloxyethyl phosphorylcholine (MPC) is a very well characterized biomedical material, which is characterized by its high resistance to non-specific protein and cell attachment and thus, in addition to its high biocompatibility, also has antithrombogenic and anti-bacterial properties (Ishihara, 2019; Woitschach et al., 2021a; Hiranphinyophat and Iwasaki, 2021). The material used in this study consisted of medical silicone as a base material coated with PMMA-MPC. One could assume that the combination of the different materials leads to different properties than a homogeneous MPC polymer.

In summary, the *in vivo* model in the presented study represents a highly adaptable and minimally invasive test system for testing different materials for their inflammatory potential in an easy-to-use manner. The test system makes it possible to quickly investigate innovative material candidates for implant manufacture with regard to their inflammatory properties and FBR. It would be conceivable to use sample bodies in this method to obtain a signal that can be evaluated more meaningfully due to the stronger interaction. Finally, the simplified model has limitations in terms of directly translating the results to humans. The human immune system is more complex than the mouse immune system (Chiappini et al., 2012). However, results from a mouse model can provide clues to potential responses in the human organism and are therefore an important component of research.

## Data Availability

The original contributions presented in the study are included in the article/Supplementary Material, further inquiries can be directed to the corresponding author.
